# The highly synergistic, broad spectrum, antibacterial activity of organic acids and transition metals

**DOI:** 10.1038/srep44554

**Published:** 2017-03-15

**Authors:** Daniel Zhitnitsky, Jessica Rose, Oded Lewinson

**Affiliations:** 1 Department of Biochemistry, The Bruce and Ruth Rappaport Faculty of Medicine, The Technion-Israel Institute of Technology, Haifa, Israel

## Abstract

For millennia, transition metals have been exploited to inhibit bacterial growth. We report here the potentiation of the anti-bacterial activity of transition metals by organic acids. Strong synergy between low, non-toxic concentrations of transition metals and organic acids was observed with up to ~1000-fold higher inhibitory effect on bacterial growth. We show that organic acids shuttle transition metals through the permeability barrier of the bacterial membrane, leading to increased influx of transition metals into bacterial cells. We demonstrate that this synergy can be effectively used to inhibit the growth of a broad range of plant and human bacterial pathogens, and suggest that a revision of food preservation and crop protection strategies may be in order. These findings bear significant biomedical, agricultural, financial and environmental opportunities.

Antibiotics are one of the keystones of modern medicine and are broadly used not only in clinical settings, but also in farm animal diets and crop protection. To date, they still present the most, and often only, effective treatment against bacterial infections. However, the success and widespread use of antibiotics is also the source of the emergence of resistance^1^. In the past decade, many bacterial pathogens have spawned strains that are highly multi-drug resistant^2^. Understandably, fatalities caused by such drug-resistant strains have fueled much commotion in the international media and have inspired apocalyptic predictions. While these reactions are perhaps disproportionate to the extent of the phenomenon, it is absolutely clear that antibiotics must be used with more restraint.

One strategy to reduce the use of antibiotics is the use of non-antibiotic alternatives whenever possible^3^. Our forefathers have used such alternatives for thousands of years. For example, to disinfect water and preserve food, copper and silver were used as early as ancient Egypt (2000 BC)^4^. It is also told that the great Persian King Cirrus (~600 B.C) refused to drink water that was not transported in silver containers. Similarly, Hippocrates, who has been referred to as the father of modern medicine, recognized the antimicrobial efficacy of transition metals and used silver-containing ointments to treat wounds^5^. The use of transition metals as antibacterial agents continues today, *e.g.,* in coating the surfaces and medical devices and in topical treatment of wounds, burns, and rashes^6^,^7^. However, their use is limited to particular settings as well as by considerations of effectiveness, cost, toxicity and possible detrimental environmental effects on soil and water reservoirs^8^.

An example of a more environmentally-friendly antibacterial alternative is the organic acid acetate: the main component of vinegar. Like transition metals, organic acids have been used for centuries to inhibit bacterial growth and carry the additional beneficial property of being non-toxic to humans. Consequently, organic acids are one of the most common food preservatives today^9^,^10^,^11^. However, relative to *bona fide* antibiotics, both transition metals and organic acids have weak antibacterial activity when used separately.

In efforts to develop safe, inexpensive and novel antibacterial approaches, we investigated the synergistic antibacterial effect of organic acids and transition metals - two broadly used and highly available resources. We resolve the underlying mechanism for the observed bacteriostatic synergism and demonstrate its effectiveness against a broad range of bacteria, including several important pathogens. In addition, we propose an application of this approach toward improving bioreactor-based production of alternative fuels.

## Results

### A surprising link between sensitivity to transition metals and sensitivity to acetate

**Figure 1:**
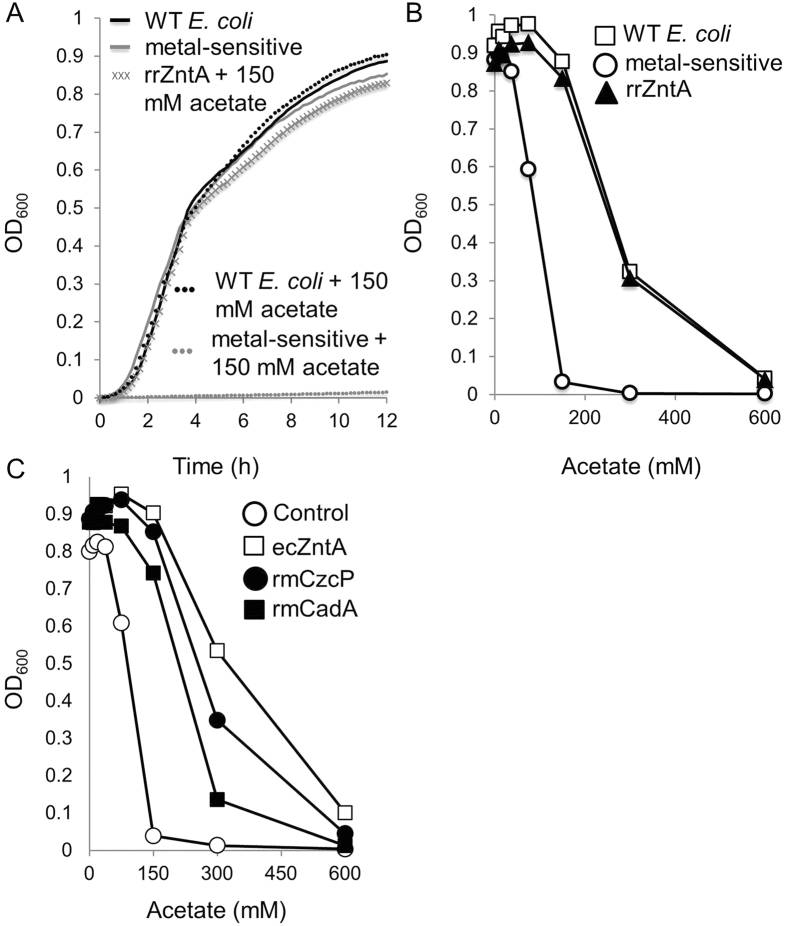
The sensitivities to acetate and transition metals are linked. (**A**) Wild type *E. coli* (black curves) or its divalent metal-sensitive derivative (grey curves) were grown for 12 hours in LB medium in the absence (solid lines) or presence (dotted lines) of 150 mM sodium acetate. The grey crosses show the growth of the divalent metal-sensitive strain (in the presence of 150 mM sodium acetate) that was transformed with a plasmid encoding the divalent metal ATPase rrZntA. (**B**) Optical density of wild-type (WT) *E. coli* cultures (squares) or its divalent metal-sensitive derivative (circles) grown for 12 hours in the absence or presence of the indicated concentrations of sodium acetate. The triangles show the growth of the divalent metal-sensitive strain that was transformed with a plasmid encoding the divalent metal ATPase rrZntA. (**C**) Same as in B, but shown are only cultures of the divalent metal-sensitive *E. coli* strain transformed with an empty control plasmid (empty circles), plasmid encoding *Ralstonia metallidurans* CadA (full squares), *Ralstonia metallidurans* CzcP (full circles) or *E. coli* ZntA (empty squares). The results in B and C are mean values (n = 3) and the standard deviations are smaller than the icons and are thus hidden.

To avoid the toxic over-accumulation of transition metals, bacteria have evolved dedicated metal efflux systems. Strains lacking such systems are acutely metal-sensitive^12^,^13^. The *E. coli* GG48 strain^12^, that carries deletions of two of its divalent metal (Zn^2+^/Cd^2+^/Pb^2+^/Hg^2+^) efflux systems (*zitB*^12^ and *zntA*^14^), exhibits very low tolerance to zinc and cadmium and to other divalent metals^13^. By serendipity, we observed that the growth of this divalent metal-sensitive strain is completely inhibited by 150 mM sodium acetate (media titrated to pH 7), while the isogenic parental strain grew to full capacity (Fig. 1A,B). The mutant strain demonstrated a similar degree of intolerance to other acetate salts, including calcium di-acetate, magnesium di-acetate, ammonium acetate, and acetic acid (not shown). In contrast, acetate-free sodium salts had no effect on growth of the mutant strain (not shown). Thus, we conclude that the divalent metal-sensitive *E. coli* strain is hypersensitive to the acetate ions in the medium.

### Expression of Zn^2+^/Cd^2+^ efflux pumps restores tolerance to organic acids

**Figure 2:**
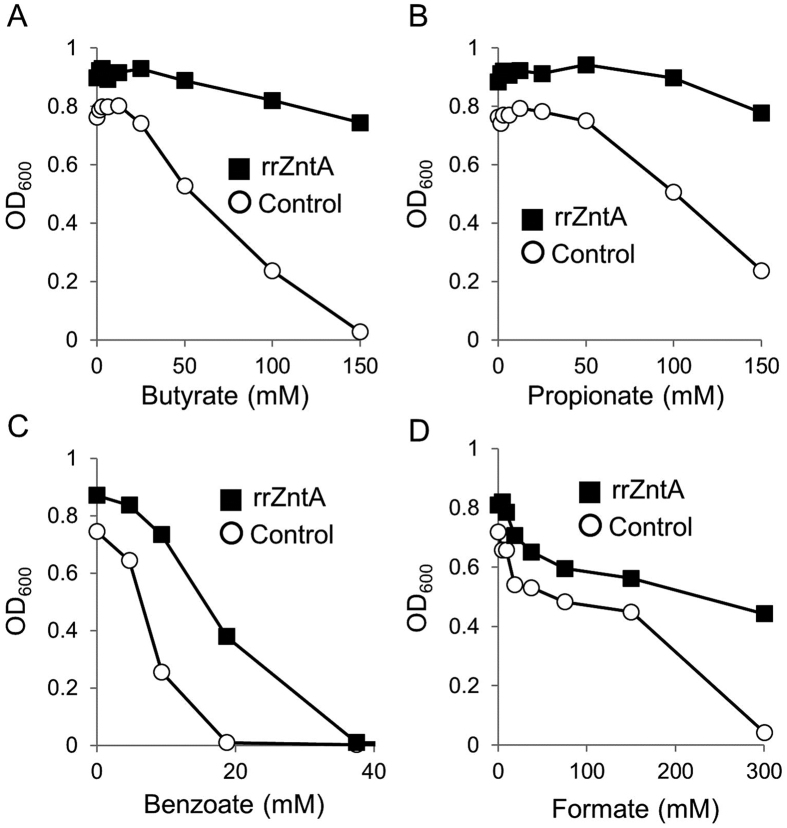
The divalent metal-intolerant strain is sensitive to a variety of organic acids. The divalent metal-sensitive *E. coli* strain was transformed with an empty control plasmid (circles) or a plasmid encoding rrZntA (squares). Cells were grown for 12 hours in LB medium in the absence or presence of the indicated concentrations of (**A**) Sodium *n*-Butyrate, (**B**) Sodium Propionate, (**C**) Sodium Benzoate, or (**D**) Sodium Formate. The results are mean values (n = 3) and the standard deviations are smaller than the icons and are thus hidden.

In an attempt to restore normal tolerance to acetate, a divalent metal-sensitive *E. coli* strain was transformed with a plasmid expressing a divalent efflux pump from *Rhizobium radiobacter* (rrZntA). We intentionally chose not to re-introduce either of the deleted *E. coli* exporters (*zitB* or *zntA*), since we wanted to focus on metal efflux rather than on regulatory roles or possible protein:protein interactions of the endogenous systems. Our previous work has shown that heterologous expression of rrZntA conveys robust metal tolerance in *E. coli*^13^. Importantly, expression of rrZntA also restored tolerance to acetate (Fig. 1A and B). Acetate tolerance was similarly restored by heterologous expression of two other Zn^2+^/Cd^2+^ efflux pumps from *Ralstonia metallidurans*^15^, and by homologous expression of the *E. coli* Zn^2+^/Cd^2+^ efflux P-type ATPase ZntA^16^ (Fig. 1C). When testing the sensitivity of the divalent metal-sensitive mutant strain to other organic acids, we found that the sensitivity of this strain is not limited to acetate. We found it was also sensitive to benzoate, formate, butyrate and propionate (Fig. 2) and expression of rrZntA restored tolerance to near wild type levels to all of these organic acids near wild type levels (not shown).

To further substantiate the correlation between tolerance of toxic concentrations of weak organic acids and metal efflux activity, the cells were transformed with mutant variants of rrZntA, that do not transport metals. The following five mutants that were characterized in the past^13^ were tested: mutant D600A, which carries a mutation in the canonical catalytic aspartic residue of P-type ATPases and cannot hydrolyze ATP, and the E366A/E381A, C556A, C558A, and D882A mutants - all deficient in metal coordination^13^,^17^. As shown in Supplementary Figure 1, unlike the robust organic acid tolerance conferred by wild-type rrZntA, all of the mutants deficient in divalent metal transport failed to restore tolerance to organic acids. When expressing a modified rrZntA featuring altered metal specificity^13^ from Zn^2+^/Cd^2+^ to Ag^+^, the bacterial cells were still intolerant to organic acids (Supplementary Figure 1). This shows that the tolerance towards organic acids can be restored only via a specific transport function and not just by any functional variant of the efflux pump.

### The mechanism for increased sensitivity towards organic acids

The toxicity of organic acids is attributed to perturbation of cellular pH homeostasis: the protonated acid diffuses into the cell where it de-protonates and alters both the ΔpH and ΔΨ^9^,^18^. We therefore hypothesized that perhaps the intolerance of the divalent metal sensitive strain to organic acids is related to pH homeostasis/tolerance. We tested this hypothesis by growing the divalent metal-sensitive strain with or without the heterologous expression of rrZntA over a range of pH (5–9). A small growth difference was observed (less than 10%) between the control and the rrZntA-expressing cells. However, this small difference remained unchanged throughout the pH range and was not amplified in either the acidic or basic end (Supplementary Figure 2A). We also found that rrZntA resistance to organic acids is independent of ΔpH, as demonstrated by experiments conducted in the presence of the proton-specific ionophore CCCP (Supplementary Figure 2B). Taken together, these results suggest that the correlation between transport of metals and sensitivity to organic acids is unrelated to pH homeostasis.

Next, we considered that organic acids may compromise the cytoplasmic membrane and therefore indiscriminately increase the cellular influx of various compounds, including the transition metals present in the growth media. Such a non-specific, detergent-like effect has previously been suggested for resin acids^19^.

To measure the effects of organic acids on membrane permeability/integrity, two methods were employed. The first method was Inductively-Coupled Plasma Mass Spectrometry (ICP-MS) and it was used to measure the effect of sodium acetate on the intracellular accumulation of ions that are generally membrane-impermeable (sodium, magnesium, calcium, potassium). Ionizing the sample with inductively coupled-plasma and then separating and quantifying the different ions using a mass spectrometer provides for a sensitive and accurate method for determining metal concentrations. As shown in Supplementary Figure 3A, a mild effect was observed with sodium and no effects were observed with magnesium, calcium or potassium. The second method was a spectroscopic assay that measures the permeability through the cytoplasmic membrane^20^,^21^. This assay measures the permeability of both the outer and the inner membranes of a permease-deficient *E. coli* strain. Only by disrupting the inner membrane of this strain can the substrate (ONPG) permeate into the cytoplasm and undergo hydrolysis. The products of said hydrolysis absorb light at 420 nm, thus allowing the monitoring of membrane disruption by different compounds and in this particular case, by organic acids. As shown in Supplementary Figure 3B, we found no evidence that organic acids generally, or indiscriminately, compromise the integrity of the cytoplasmic membrane. These combined results argue against a non-specific effect of organic acids.

**Figure 3:**
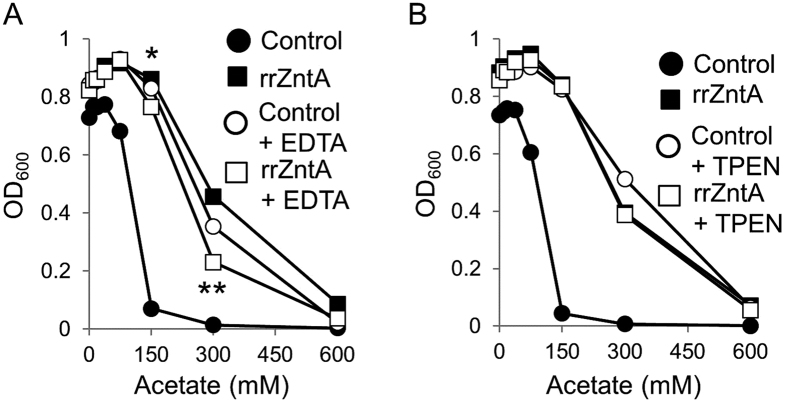
Metal chelates alleviate the sensitivity to organic acids. (**A**) The divalent metal-sensitive *E. coli* strain was transformed with an empty control plasmid (circles) or a plasmid encoding rrZntA (squares). Cells were grown for 15 hours in LB medium in the presence of the indicated concentrations of sodium acetate and in the absence (full symbols) or presence (open symbols) of 0.2 mM EDTA (**A**) or 15 μM TPEN (**B**). The results are mean values (n = 3) and the standard deviations are smaller than the icons and are thus hidden.

Organic acids are known to form complexes with transition metals (*e.g.,* zinc-acetate, copper-acetate and copper-butyrate^22^,^23^,^24^). The growth experiments described above were conducted at physiological pH at which the organic acids are almost entirely de-protonated and negatively charged. Potentially, under these conditions, they can form electro-neutral complexes with the positively-charged transition metals that are present in growth media. Formation of such electro-neutral complexes would facilitate the permeation of both organic acids and transition metals and would explain the observed toxicity. To test this hypothesis, growth experiments were repeated in chemically-defined medium that contained lower levels of transition metals than complex media. As shown, when grown in minimal medium, the growth difference between the divalent metal-sensitive and WT strains was smaller than the difference observed in LB (compare Supplementary Figure 4 to Fig. 1). Upon introduction of the non-specific metal chelator EDTA, the organic-acid sensitivity of the divalent metal-sensitive strain was fully alleviated to a point where it grew better than the same strain expressing rrZntA (Fig. 3A). This growth disadvantage of the rrZntA-expressing cells likely stems from the intracellular over-depletion of zinc caused by the unregulated activity of rrZntA, combined with the scarcity of free zinc in the media. Similar results were obtained when adding the zinc-specific chelator TPEN to the medium (Fig. 3B). Together, these results (Fig. 3 and Supplementary Figure 4) demonstrate that the sensitivity to organic acids is directly proportional to the concentration of metals in the medium where high sensitivity is proportional to metal abundance, and low sensitivity is proportional to metal depletion.

**Figure 4:**
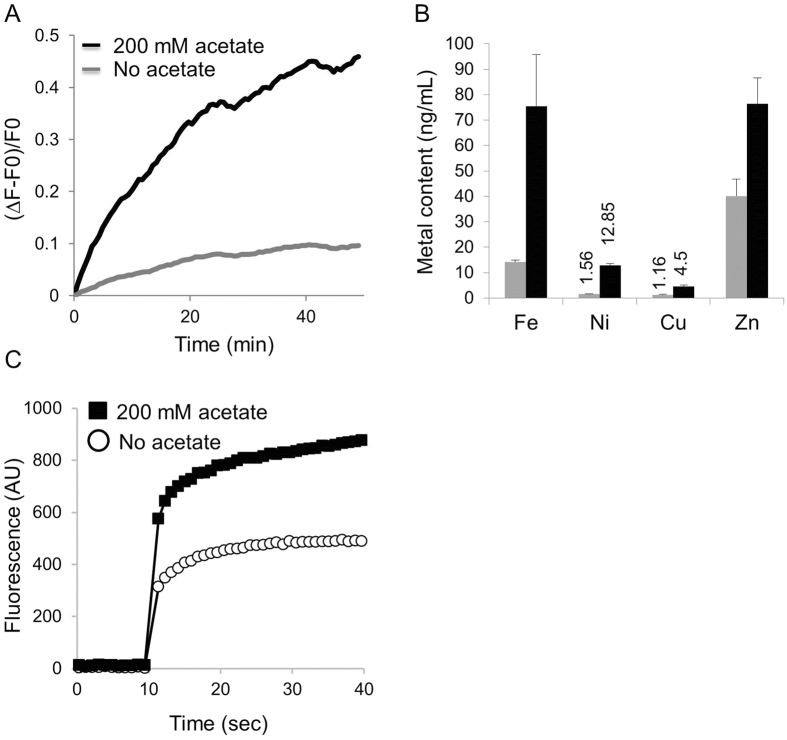
Organic acids increase influx and intracellular accumulations of metals. (**A**) Spheroplasts prepared from the divalent metal-sensitive *E. coli* strain were loaded with 20 μM of the Zn-specific fluorescent dye Zinquin. At time zero, 1 mM ZnSO_4_ was added to the cells in the absence (grey curve) or presence (black curve) of 200 mM sodium acetate. Shown is the Zinquin-associated fluorescence that was continuously measured. (**B**) The metal content of *E. coli* cells was determined by ICP-MS following growth in LB medium in the absence (grey bars) or presence (black bars) of 50 mM sodium acetate. (**C**) Liposomes were loaded with 40 μM Zinquin, and after 10 seconds ZnSO_4_ (20 μM) was injected in the absence (open circles) or presence (black squares) of 200 mM sodium benzoate.

We measured the rate of cellular influx of transition metals in the absence or presence of organic acids. To do this, the bacteria were loaded with Zinquin: a specific fluorescent indicator of zinc, and metal influx was monitored in real time. As shown in Fig. 4A, acetate accelerated zinc influx by 3 to 4-fold. To assess whether the increased metal influx was specific to zinc or common to other transition metals, cells were grown in rich medium (LB) in the absence or presence of 50 mM sodium acetate and intracellular metal levels were determined by ICP-MS. This analysis revealed higher levels of zinc, copper, nickel and iron in the cells grown in the presence of sodium acetate (Fig. 4B). The greatest effects were on Ni and Fe that accumulated to ~8 and 5 to 6-fold higher intracellular concentrations in the presence of sodium acetate, respectively (Fig. 4B). To test whether the increased influx/accumulation of transition metals depended on the cellular context, liposomes from *E. coli* lipids were generated and loaded with Zinquin. In this cell-free system the rate of zinc influx accelerated in the presence of organic acids, and higher final intra-liposomal levels were obtained (Fig. 4C). Collectively, these results demonstrate that organic acids increase the membrane permeability of metals consequently leading to elevated intracellular metal accumulation.

### Synergy of bacterial inhibition between organic acids and metals

The increased metal influx mediated by organic acids predicts that the bacteriostatic effects of organic acids and transition metals are not additive, but synergistic. To assess and quantify synergy, two independent approaches were used: the Bliss independence model^25^,^26^,^27^ and the Median-Effects model^28^,^29^,^30^. The Bliss independence model was used due to its intuitive nature and simplicity. To calculate the Bliss values, the cells were first grown in the presence of the individual inhibitors. Then, under the assumption that the compounds have no interaction, the expected inhibition was calculated by simply finding the product of the individual fractional inhibitions (Supplementary Figure 5). Expected/observed growth ratios of ~1 indicate an additive effect, while larger values indicate synergy. We considered expected/observed growth ratios larger than 20 to be significant (see example in Supplementary Figure 5). As an additional approach to quantify synergy we used the Median-Effects model which is broadly applied to assess and to measure pharmacological synergistic effects in drug combination experiments^31^. The Median-Effects model combines the Michaelis-Menten equation of enzyme kinetics, the Hill equation for higher-order ligand binding saturation, the Henderson-Hasselbalch equation for pH ionization and the Scatchard equation for receptor binding^28^,^29^,^30^ for the calculation of Combination Indices (CI values). Calculated directly from the Median Effects model, the CI values quantitatively define synergism (CI < 1), additive effect (CI = 1) and antagonism (CI > 1). The two methods for assessing synergy use completely different methodologies and are thus independent from one another. Identification of synergistic affects with both methods lends strong support to any claim of synergy.

**Figure 5:**
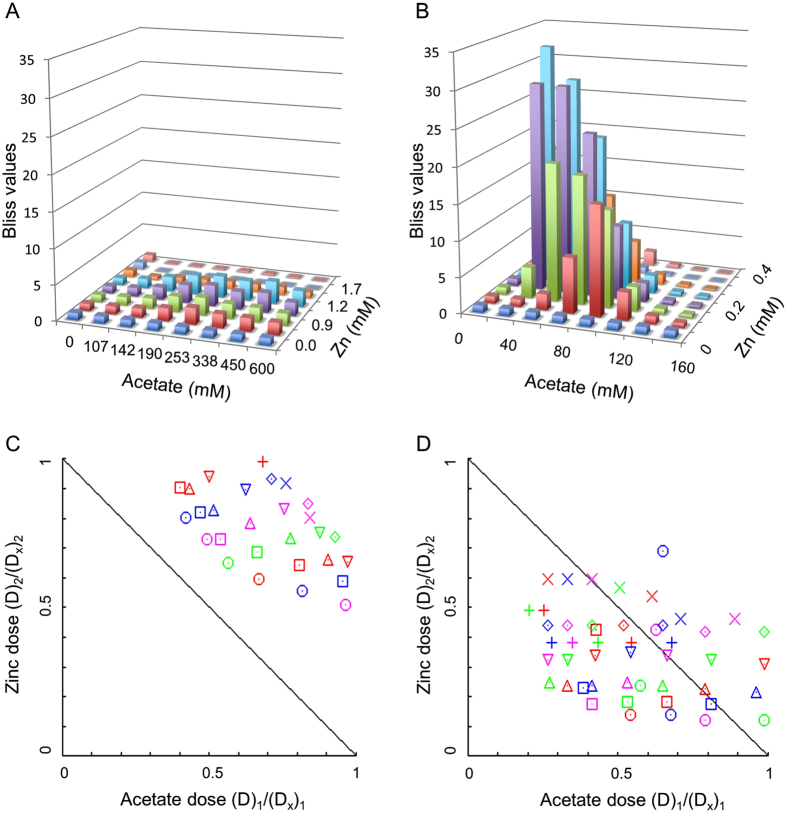
The link between metal and acetate sensitivities indicates synergy. Bliss (**A**,**C**) and CI (**C**,**D**) values were calculated for WT (**A**,**C**) and divalent metal-sensitive (**B**,**D**) bacteria that were grown for 15 hours in an 8 × 8 matrix at the indicated concentrations of sodium acetate and ZnSO_4_. (**C**) Isobologram generated using *Compusyn* software for the 8 × 8 matrix shown in A yielding R-values of R = 0.93 for zinc data and R = 0.98 for sodium acetate data (**D**) Isobologram generated using *Compusyn* software for the 8 × 8 matrix shown in B yielding R-values of R = 0.93 for zinc data and R = 0.99 for sodium acetate data.

Figure 5 shows the application of both models to the sensitivity of WT *E. coli* and to the divalent metal-sensitive strain to 64 combinations of acetate and zinc. With both models it is very clear that in WT *E. coli* there is little to no synergy (Fig. 5A,C). This is to be expected since WT *E. coli* successfully cope with the increased influx of zinc. In contrast, synergy was clearly observed in the case of the divalent metal-sensitive strain (Fig. 5B,D). The distribution of Bliss values classically manifested synergy^32^: Bliss values near 1 at the very low or high concentrations, and high Bliss values in the intermediate concentrations (Fig. 5B). Similarly, the majority of the tested combinations were found below the diagonal of additivity of the Combination Indices isobologram, indicating synergy (Fig. 5D).

**Figure 6:**
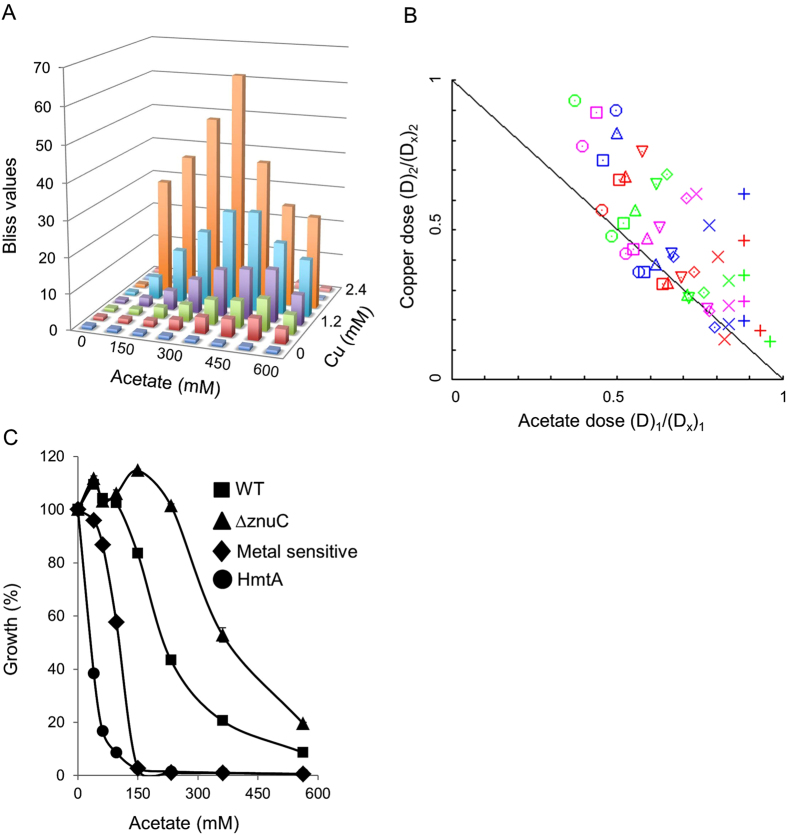
Inhibition of WT *E. coli* by combinations of copper and acetate. (**A**) Bliss values were calculated for WT *E. coli* that were grown for 15 hours in an 8 × 8 matrix of the indicated concentrations of sodium acetate and CuSO_4_. (**B**) Isobologram generated using *Compusyn* software for the 8 × 8 matrix described in A yielding R-values of R = 0.98 for zinc data and R = 0.97 for sodium acetate data. (**C**) Manipulating the sensitivity/tolerance to acetate by changing the metal influx/efflux balance. Cultures of WT *E. coli* (squares), the Zn-uptake deficient *E. coli* strain (Δ*znuC,* triangles), the divalent metal-sensitive strain (diamonds), or cells expressing the *P. aeruginosa* metal import pump HmtA (circles) were grown for 15 hours in the absence or presence of the indicated concentrations of sodium acetate. Growth in the absence of sodium acetate was defined as 100%. The results are mean values (n = 3) and the standard deviations are shown.

The ICP-MS analysis (Fig. 4B) revealed that in addition to zinc, the influx of copper is also accelerated by acetate. The synergistic inhibitory effects of copper and acetate on the growth of WT *E. coli* were subsequently examined. As shown in Fig. 6A and B, the synergy between acetate and copper was even stronger than with zinc with a maximal Bliss value of 63. In light of this synergy, and the observed differences between WT *E. coli* and the divalent metal-sensitive strain, we reasoned that the tolerance of the bacteria to one agent can be changed by manipulating their tolerance to another. Cellular influx/efflux of zinc was manipulated in two ways. The first way involved using a *znuC*-deleted *E. coli* strain to demonstrate reduction in zinc influx. *ZnuC* is the trans-membrane domain of *znuABC* which is the main zinc import system in *E. coli*^33^. The second way involved the transformation of cells using a plasmid expressing the *Pseudomonas aeruginosa* metal importer HmtA (HmtA leads to acute metal hypersensitivity^34^) to demonstrate an increase in metal influx to the cells. As shown in Fig. 6C, the sensitivity of *E. coli* cells to acetate was directly correlated with their metal influx/efflux balance. With its normal metal uptake/efflux activity, WT *E. coli* serves as the reference in this experiment. In the Δ*znuC* strain, the reduced metal uptake combined with the normal metal efflux led to increased tolerance of acetate. In contrast, as demonstrated by the *E. coli* cells expressing the plasmid-borne metal importer HmtA, increasing their metal import activity resulted in acute hypersensitivity towards acetate. These results, combined with the reduced toxicity of organic acids in the presence of metal chelators (Fig. 3), demonstrate a direct correlation between tolerance/sensitivity to organic acids and efflux/influx of metals.

### Synergistic inhibition of a range of bacterial pathogens

**Figure 7:**
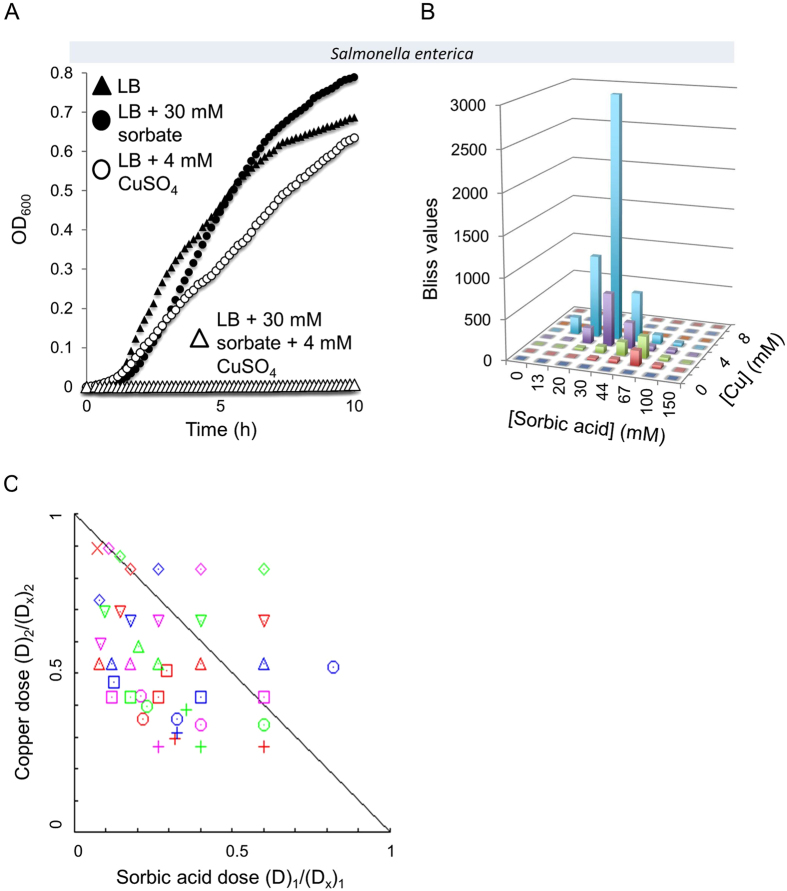
Synergistic growth inhibition of *Salmonella enterica*. (**A**) Cultures of *Salmonella enterica* serovar typhimurium were grown in LB medium (closed triangles), LB medium supplemented with 30 mM sorbic acid (closed circles), LB medium supplemented with 4 mM CuSO_4_ (open circles) or supplemented with both 30 mM sorbic acid and 4 mM CuSO_4_ (open triangles). (**B**) Bliss values were calculated for *Salmonella enterica* serovar typhimurium that was grown for 15 hours in an 8 × 8 matrix of the indicated concentrations of sorbic acid and CuSO_4_. (**C**) Isobologram generated using *Compusyn* software for the 8 × 8 matrix described in B yielding R-values of R = 0.99 for sorbic acid data and R = 0.95 for copper data.

In order to evaluate the potential of the synergistic effects between organic acids and transition metals to inhibit bacteria other than *E. coli*, inhibition of three human bacterial pathogens, *Salmonella enterica* (ATCC 14028), *Pseudomonas aeruginosa* (PA01) and *Vibrio cholerae* (B1) were tested. *Salmonella enterica* is a facultative intracellular pathogen and is one of the most common food-transmitted bacterial pathogens. By itself, the commonly-used food preservative sorbic acid is a poor inhibitor of *Salmonella* growth, as is the dietary supplement copper sulfate (Fig. 7A). However, when combined, these two agents showed a remarkable ability to inhibit *Salmonella* growth indicating a strong synergistic effect. Bliss values were very high at ~3000, and 62% of all CI values were indicative of synergy (CI < 1) (Fig. 7B and C, respectively), which practically means zero bacterial growth (Fig. 7A). The growth of *Salmonella* was also very effectively inhibited by benzoic acid (another food preservative) when combined with copper (Supplementary Figure 6A,B). *Pseudomonas aeruginosa* is a common cause of nosocomial infections and is notorious for its high level of antibiotic resistance. Low concentrations of either copper or acetic acid ineffectively inhibited the growth of *P. aeruginosa,* yet their combination has shown a very high antibacterial synergy (Supplementary Figure 6C,D). The growth of the aquatic pathogen *Vibrio cholerae* was also modestly inhibited by individual challenges of either transition metals or organic acids, yet very effectively inhibited by their combinations (Supplementary Figure 7A,B). The experiments presented so far were all conducted with Gram-negative bacteria. To test whether the synergy between transition metals and organic acids can also be used to inhibit the growth of Gram-positive bacteria, similar experiments were conducted with the model bacterium *Bacillus subtilis* (ATCC 23857). As shown in Supplementary Figure 7C and D, this Gram-positive bacterium was also susceptible to combinations of transition metals and organic acids.

**Figure 8:**
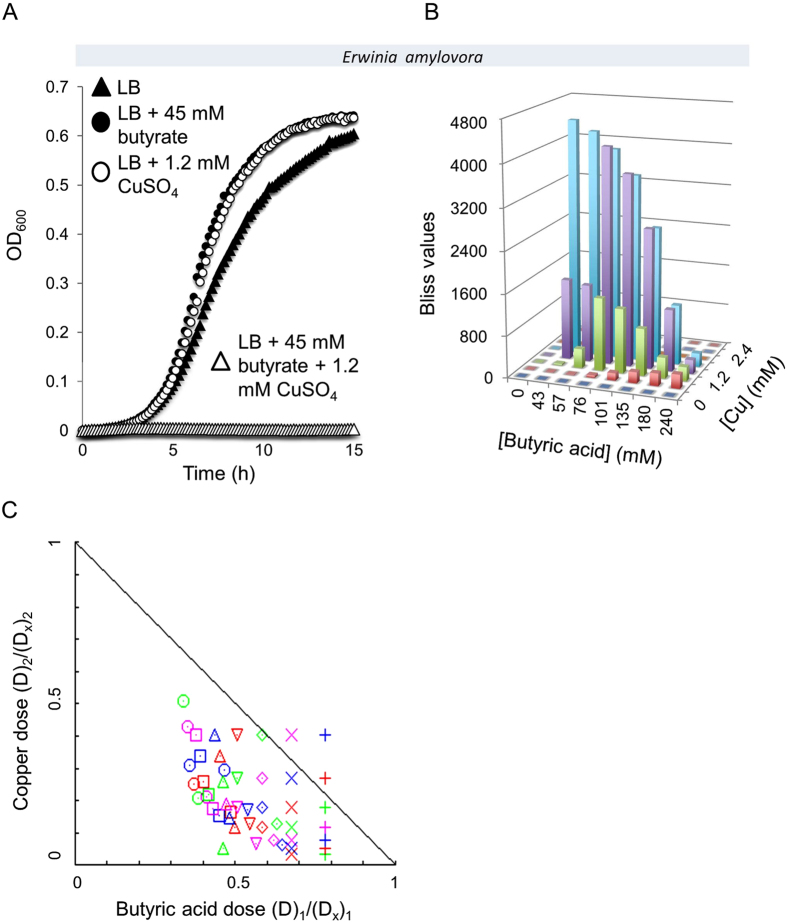
Synergistic growth inhibition of the bacterial plant pathogen *Erwinia amylovora*. (**A**) Cultures of *Erwinia amylovora* were grown in LB medium (closed triangles), LB medium supplemented with 45 mM butyric acid (closed circles), LB medium supplemented with 1.2 mM CuSO_4_ (open circles) or supplemented with both 45 mM butyric acid and 1.2 mM CuSO_4_ (open triangles). (**B**) Bliss values calculated for *Erwinia amylovora* that was grown for 15 hours in an 8 × 8 matrix of the indicated concentrations of butyric acid and CuSO_4_. (**C**) Isobologram generated using *Compusyn* software for the 8 × 8 matrix described in B yielding R-values of R = 1 for butyric acid data and R = 0.91 for copper data.

Bacterial plant pathogens are a serious threat to the global food supply^35^ and crops are routinely treated with antibacterial agents^36^,^37^,^38^. One such very common treatment is copper spray^39^,^40^. In light of our previous synergy results, we tested if the efficacy of this treatment could be enhanced by the addition of selected organic acids. Three pathogens of great agricultural burden were tested: *Erwinia amylovora* (209), *Pseudomonas syringae* (ATCC BAA-871) and *Xanthomonas euvesicatoria* (85–10). The growth of *Erwinia amylovora* was unaffected when only copper was added to the growth media and similar results were obtained with butyric acid. However, when these agents were combined, complete growth arrest of *Erwinia amylovora* was achieved (Fig. 8A). This inhibition was highly synergistic with Bliss values as high as ~4700 and 92% of all CI values indicative of synergy (Fig. 8B and C, respectively). The two other plant pathogens, *Pseudomonas syringae* and *Xanthomonas euvesicatoria* were also highly sensitive to combinations of organic acids and transition metals (Supplementary Figure 8).

## Discussion

The worldwide rise in bacterial infection-associated morbidity underscores the need to implement novel approaches to limit bacterial growth^41^. Antibiotics remain the treatment of choice but their excessive and injudicious use has led to high incidences of drug and multidrug-resistant bacterial strains^42^. Moreover, agricultural use of antibiotics and bactericides results in environmental damage and can lead to widespread and unpredicted harm to wildlife^43^. It is therefore crucial to find alternatives to conventional antibiotics. In this work we present an inexpensive and viable alternative by simply using combinations of compounds that have relatively low toxicity to humans, animals, and plants. This particular approach is simple, readily applicable and effective. Organic acids are perhaps the most common food preservatives and it would be very simple to add zinc or copper (ordinary dietary supplements) as an additional preservative.

We showed that the common food-borne pathogen *Salmonella enterica* is poorly inhibited by both organic acids and transition metals, yet completely inhibited by their combination. This was also true for all the other bacterial species that we tested (including pathogens such as *Pseudomonas aeruginosa* and *Vibrio cholerae*). We therefore suggest that food preservation can be improved by exploiting the synergistic effect of combining simple organic acids and metals. A similar revision to strategies to protect crops is suggested. World-wide, copper is routinely sprayed on crops, orchards and flowers^40^. It is often the only available preventive measure in places where antibiotics sprays are banned. However, the copper doses are limited by their toxicity towards plants^44^ and the efficacy of the sprays are compromised by the emergence of copper-resistant bacterial strains^45^. We have consistently observed that copper readily combines with all tested organic acids to form a highly potent bactericide that inhibits the growth of the plant pathogens *Erwinia amylovora, Pseudomonas syringae* and *Xanthomonas euvesicatoria*. Copper sprays are non-aqueous emulsions that contain fatty acids and other organic compounds and therefore addition of organic acids to these emulsions is straightforward. This sets the stage for a fast, easy and safe transition to a better way to ensure bactericidal effects with limited toxicity to the plants and ecosystems alike.

It is vital to consider the effect that combinations of metals and organic acids may have on the environment as well as on plant or animal cells. The synergistic nature of the inhibitory effect allows the use of much lower concentrations of organic acids and of metals than those used for each of them individually. Thus, the distribution of metal pollutants from crop sprays as well as concentrations of food preservatives may actually decrease by combining organic acids with metals. Furthermore, an adult human can tolerate digestion of up to 10 mg of copper and up to 40 mg of zinc per day^46^, while copper sprays of potato plants, for example, contain 2.5 grams/liter concentrations of copper and can tolerate up to 5 grams/liter^44^. Moreover, synergy growth experiments were performed on two eukaryotic microorganisms, *Saccharomyces cerevisiae* and *Candida albicans*, with the following combinations: acetate-copper, acetate-zinc, benzoate-zinc and benzoate-copper. Benzoate was selected since it potentially forms the most hydrophobic complex with metals, thus should have a high synergistic effect (if one is present). In most cases, little to no synergy was observed, with maximal Bliss values of >5 (not shown). For *Candida albicans* with the benzoate-copper combination however, a maximal Bliss value of ~175 was observed (not shown). The R-values for this combination were at 0.78–0.79 for both copper and benzoate, indicating a low statistical fit to the Median-Effects model. Thus, despite the clear need for further study, based on this data and on our results we believe that the suggested revisions to crop protection and food preservation are likely safe.

These results may also be relevant in the context of bacterial growth promotion. One such example relates to current efforts to replace fossil fuels with biofuel alternatives. Biofuel alternatives rely on bacterial organic acid production in bioreactors^47^,^48^ which is currently limited by bacterial growth inhibition due to accumulation of the desired organic acid product^49^. Our data suggest that simple addition of a metal chelator may increase the commercial efficiency of bioreactor production of alternative fuels. In addition, bacterial strains with improved metal efflux activity (*e.g.,* via over-expression of metal efflux pumps) are promising commercial producers of organic acids.

In summary, organic acid and transition metal combinations synergistically inhibit the growth of a broad range of bacteria including growth of several important pathogens. Application of such combinations may provide a cost-effective, simple and efficient alternative to current bactericidal and antibiotic agents. We believe that these results are relevant to any setting where microbial growth control is of essence.

## Materials and Methods

### Bacterial growth

Unless otherwise indicated, bacteria were grown in LB medium (10% NaCl, 10% Tryptone, and 5% Yeast extract). Cultures were grown at 37 °C (*E. coli, B. subtilis, P. aeruginosa, V. cholerae,* and *S. enterica*), or 28 °C (*P. syringe, X. euvesicatoria* and *E. amylovora*). To manipulate the pH of the media, it was titrated with NaOH and HCl, and verified with a pH-meter.

### Organic acid sensitivity assays

Cultures were diluted (96-well plates) to an OD_600_ of 0.05 in 150 μl of the media in the presence of the indicated concentrations of sodium organic acid salts and/or transition metals. Where applicable, L-Arabinose (0.02%) was included in the growth media to induce low expression of plasmid encoded transporters. Growth was monitored continuously for 12–15 hours in an automated plate reader (Infinite M200 pro, Tecan). Growth with EDTA (Ethylenediaminetetraacetic acid), TPEN (*N’,N’,N’,N’*-tetrakis-(2-pyridylmethyl)ethylenediamine), or CCCP (Carbonyl cyanide *m*-chlorophenyl hydrazine) was perform in the same manner, but with a pre-determined (indicated) sub-lethal concentration of the chelators/protonophore.

### Spheroplast preparation and measurements of zinc uptake

25 ml of divalent metal-sensitive *E. coli* were grown in LB to an OD_600_ of 0.3–0.5 and harvested. The cells were then washed with 2.5 ml of ice-cold 10 mM Tris‐HCl pH 7.5 and 0.75 M sucrose buffer. The cells were re-suspended in the same buffer, with 0.1 mM Lysozyme, 1.25 mM EDTA, 25 mM MgCl_2_ and DNase. After a 30-minute incubation, the spheroplasts were centrifuged at 2,000 × g for 10 min at 4 °C and re-suspended in 50 mM Tris‐HCl pH 7.5, 150 mM NaCl, 100 μg/ml DNase, and 5 mM MgCl_2_. 20 μM of Zinquin ethyl ester salt was added, and the spheroplasts were incubated, with gentle shaking, in 37 °C for 30 minutes. The spheroplasts were then washed with 50 mM Bis-Tris pH 6, 150 mM NaCl at 4 °C and re-suspended in the same buffer. The spheroplasts were then dispensed in triplicates into black 96-well plates to an OD_600_ of 0.1 in 150 μl of the buffer, in the presence or absence of the indicated concentrations of acid-sodium salts. Fluorescence was monitored continuously in an automated plate reader (Infinite M200 pro, Tecan) with excitation at 368 nm and emission at 490 nm. The assay was initiated by injecting 1 mM of ZnSO_4_.

### Cellular metal content determination by ICP-MS

Cells were grown in LB and diluted to an initial OD_600_ of 0.05 in LB containing the indicated concentrations of acetate and/or ZnSO_4_. Cells were then grown for 6–8 hours and 1 ml was harvested for each sample. Cells were then washed once with ice-cold 50 mM Tris·HCl (pH 7.5) + 100 mM KCl (HPLC-grade water) and re-suspended in 1 ml 70% Nitric acid. The samples were incubated at 100 °C until the acid vaporized completely and re-suspended in 2 ml 3% Nitric acid. The samples were analyzed at The Fredy & Nadine Herrmann Institute of Earth Sciences at the Hebrew University in Jerusalem, Israel.

### Measurements of zinc uptake in liposomes

20 mg L-α-PC and 60 mg *E. coli* polar lipids were mixed in a pre-cleaned Corex tube. A thin film was created under N_2_ stream, and desiccated overnight. The lipids were then re-suspended to 10 mg/ml with N_2_ flushed MES (pH 6.5) + 150 mM NaCl (RT) and flash-frozen in liquid nitrogen and stored at −80 °C until use. To prepare the liposomes, 10 mg of lipids were thawed at room temperature and ultra-centrifuged at 4 °C 186,000 g for 20 minutes. The lipids were then washed once with N_2_ flushed MES (pH 6.5) + 150 mM NaCl and 40 μM Zinquin were added. After a brief sonication in a bath sonicator, the lipids were extruded through a 400 nm polycarbonate membrane using a Mini-Extruder (Avanti Polar Lipids) 11 times and ultra-centrifuged at 4 °C 186,000 g for 20 minutes. The liposomes were then washed once with MES (pH 5.5) + 150 mM NaCl and re-suspended to 10 mg/ml for immediate use. The liposomes were dispensed in triplicates into black 96-well plate with the indicated concentrations of detergent (1% Triton X-100) or acid-sodium salt. Fluorescence was monitored continuously in an automated plate reader (Infinite M200 pro, Tecan) with excitation at 368 nm and emission at 490 nm. The assay was initiated by injecting 20 μM of ZnSO_4_.

### Growth assays

The assays were performed as the organic acid sensitivity assays were. The 96-well plates were prepared as a two-dimensional matrix of different organic acids and metal salts concentrations. The metal salts used were ZnSO_4_ and CuSO_4_ and growth was monitored continuously for 15–20 hours (depending on the bacterial growth rate) in an automated plate reader (Infinite M200 pro, Tecan).

### Membrane permeability assay

The assay was performed as previously described^20^,^21^,^50^. Briefly, ML-35p *E. coli* cells were grown in Tryptic soy broth (TSB) medium at 37 °C to O.D_600_ ~1. The cells were washed 3 times with SPB (10 mM NaH_2_PO_4_, pH 7.4) buffer. The cells were then diluted to OD_600_ of 0.1 in SPB buffer with 3% TSB at pH 6.4. The cells were dispensed into wells of a 96-well plate (100 μl/well) containing 200 mM of organic acids or 0.25% Triton X-100 and 2.5 μM *ortho*-nitrophenyl-β-galactoside (ONPG). Absorption at 420 nm was monitored over time in an automated plate reader (Infinite M200 pro, Tecan).

### Calculations of Bliss values

Expected results were calculated from the control groups that were grown without acid or metal under the assumption that the organic acids and the metals had only additive effects (no synergy). Bliss values were calculated as the ratio between the expected (calculated) growth and the observed growth for each cell in the matrix. See Supplementary Figure 5 for an example of such calculations.

### Calculations of Combination Indices

Combination indices were calculated using *Compusyn* software to determine the level of antagonism or synergism between chosen metals and acids. Data used for the analysis were input as inhibitory effects on bacterial growth arising from the combination of a series of increasing concentrations of metals and acids. Inhibition was calculated as the percentage optical density relative to a negative control. A combination index (CI) < 1 indicated a synergistic effect, CI = 1 indicated an additive effect and CI > 1 indicated an antagonistic effect. Combination indices are quantifications of the effects of combining drugs as calculated by using the Median-Effect equation, which merges the Michaelis-Menten equation of enzyme kinetics, the Hill equation for higher-order ligand binding saturation, the Henderson-Hasselbalch equation for pH ionization and the Scatchard equation for receptor binding^28^,^29^,^30^ thus utilizing mass-action laws and mathematical induction-deduction. Isobolograms were also generated using *Compusyn* software and illustrate the map of all possible CIs as per data point in the field described by the combined doses and effects. The calculation of the median effect returns an *R*-value that determines how well the data fits the mass-action law of the model (*R* = 1 indicates a perfect fit). In all experiments R-values greater than 0.9 are considered acceptable. Unless otherwise stated n represents the number of replicates for every experiment.

## Additional Information

**How to cite this article:** Zhitnitsky, D. *et al*. The highly synergistic, broad spectrum, antibacterial activity of organic acids and transition metals. *Sci. Rep.* **7**, 44554; doi: 10.1038/srep44554 (2017).

**Publisher's note:** Springer Nature remains neutral with regard to jurisdictional claims in published maps and institutional affiliations.

## Supplementary Material

Supplementary Figures
