# Supplementary material for: The highly synergistic, broad spectrum, antibacterial activity of organic acids and transition metals
Source: Sci Rep. 2017 Mar 15;7:44554. doi: 10.1038/srep44554 (PMC5353632; doi:10.1038/srep44554)
Supplement: Supplementary Figures [file srep44554-s1.pdf]

The highly synergistic, broad spectrum, antibacterial activity of organic acids and transition metals.

Daniel Zhitnitsky, Jessica Rose and Oded Lewinson

Department of Biochemistry,

The Bruce and Ruth Rappaport Faculty of Medicine,

The Technion-Israel Institute of Technology,

Haifa, Israel

Corresponding author: Oded Lewinson

e-mail: lewinson@tx.technion.ac.il; telephone: +972-4-8295428; fax: +972-4-8295205

SUPPLEMENTARY FIGURE LEGENDS

Supplementary Figure 1. Transport-deficient mutants of rrZntA do not restore tolerance to organic acids. The divalent metal-sensitive *E. coli* strain *(ΔzitB*, *ΔzntA*) was transformed with an empty control plasmid (red) or with a plasmid encoding WT rrZntA (black) or its mutant variants E366A/E381A (grey), C556A (blue), D882A (magenta), D600A (green) or the Ag^+-specific variant (cyan). The latter three curves overlay the red curve and cannot be distinguished. Cells were grown for 12 hours in LB medium supplemented with 150 mM sodium acetate.

Supplementary Figure 2. Acetate resistance is not related to pH resistance nor is it ΔpH dependent. (A) Divalent metal-sensitive *E. coli* expressing rrZntA (squares) or transformed with a control vector (circles) were grown for 12 hours in LB at the indicated pH values. Shown are mean results of triplicates (n=3), and error bars represent standard deviations. (B) Divalent metal-sensitive *E. coli* expressing rrZntA (squares) or transformed with a control vector (circles) were grown in LB medium for 15 hours in the presence (open symbols) or absence (full symbols) of 10 μM Carbonyl cyanide *m*-chlorophenyl hydrazine (CCCP) in the indicated acetate concentrations. Growth was monitored by measurement of the optical density at 600 nm.

Supplementary Figure 3. Organic acids do not disrupt the *E.coli* inner membrane.

(A) Wild-type *E. coli* W3110 were grown for 6 hours in LB containing 75 mM (White), 100 mM (grey) or in the absence (black) of acetate. Shown is the percentage of change in ion content, based on ICP-MS analysis results without acetate, as a baseline (100%). (B) *E. Coli* ML35p cells were incubated in phosphate-buffered saline in the absence (green) or presence of 200 mM

organic acids (acetate - purple, benzoate – cyan, butyrate – orange, formate – violet or propionate – rose) or 0.25% Triton X-100 (red) as a positive control, or buffer without cells (black) as a negative control. Absorption at 420 nm of ONPG was monitored as an indicator of inner membrane permeability. Shown are mean results of triplicates (n=3), and error bars represent standard deviations.

Supplementary Figure 4. The intolerance to organic acids is partially maintained in minimal media. The divalent metal-sensitive *E. coli* strain (Δ*zitB*, Δ*zntA*) was transformed with an empty control plasmid (circles) or a plasmid encoding rrZntA (squares). Cells were grown for 15 hours in Davis minimal medium in the presence of the indicated concentrations sodium acetate. The results are mean values (n=3) and error bars are shown unless smaller than icons and represent the standard deviations.

Supplementary Figure 5. Calculations of Bliss values. Shown is a theoretical result of a growth experiment. The growth in the absence of inhibitors is defined as 100%. In this example, in the presence of the tested concentration of inhibitor A, the cells grow to 90% of full capacity. In the presence of the tested concentration of inhibitor B, the cells grow to 85% of full capacity. Assuming additive effects only, the expected growth in the presence of A+B is 0.90 x 0.85/100= 0.765, *i.e.*, 76.5%. However in this example, the actual growth that was observed experimentally is only 2.5% of full capacity. Therefore, the expected/observed ratio (or "Bliss value") for these specific concentrations of A&B is 76.5/2.5 = 30.6. This calculation is made for each combination of A&B (in our case A&B are an organic acid and a metal). Each result, or "Bliss value", is one column in the 3-D graphs shown in Figures 5 - 8, and in supplementary Figure 6.

Supplementary Figure 6. Synergistic Inhibition of *Salmonella enterica* and *Pseudomonas aeruginosa.* Cultures of *Salmonella enterica* (A and B) or *Pseudomonas aeruginosa* (C and D) were grown in LB media in the absence or presence of the indicated concentrations of $CuSO_4$ and/or an organic acid, as indicated. Panels A and C show an example of the optical density at 600 nm after growth for 15 hours, for a single combination of $CuSO_4$ and an organic acid. Panels B&D show the calculated Bliss values for all of the tested combinations.

Supplementary Figure 7. Synergistic Inhibition of *Vibrio cholerae* and *Bacillus subtilis.* Cultures of *Vibrio cholerae* (A and B) or *Bacillus subtilis* (C and D) were grown in LB media in the absence or presence of the indicated concentrations of $CuSO_4$ or $ZnSO_4$ and/or an organic acid as indicated. Panels show an example of the optical density at 600 nm after growth for 15 hours for a single combination of copper (or zinc) and an organic acid.

Supplementary Figure 8. Synergistic Inhibition of *Pseudomonas syringae* and *Xanthomonas euvesicatoria.* Cultures of *Pseudomonas syringae* (A and B) or *Xanthomonas euvesicatoria* (C and D) were grown in LB media in the absence or presence of the indicated concentrations of $CuSO_4$ and/or an organic acid as indicated. Panels show an example of the optical density at 600 nm after growth for 15 hours for a single combination of $CuSO_4$ and an organic acid.

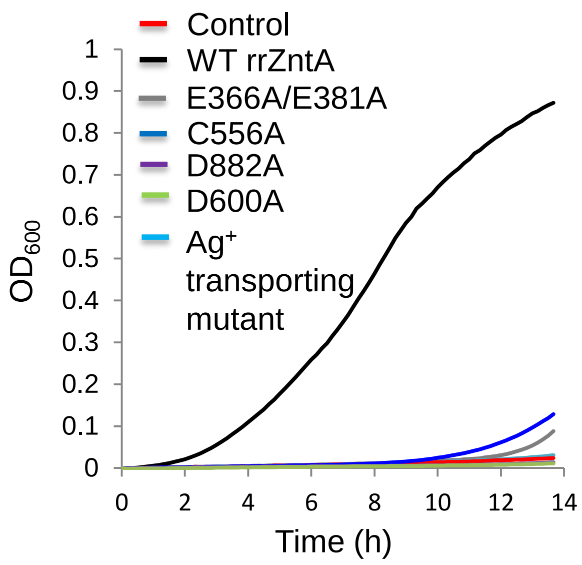

Supplementary Figure 1

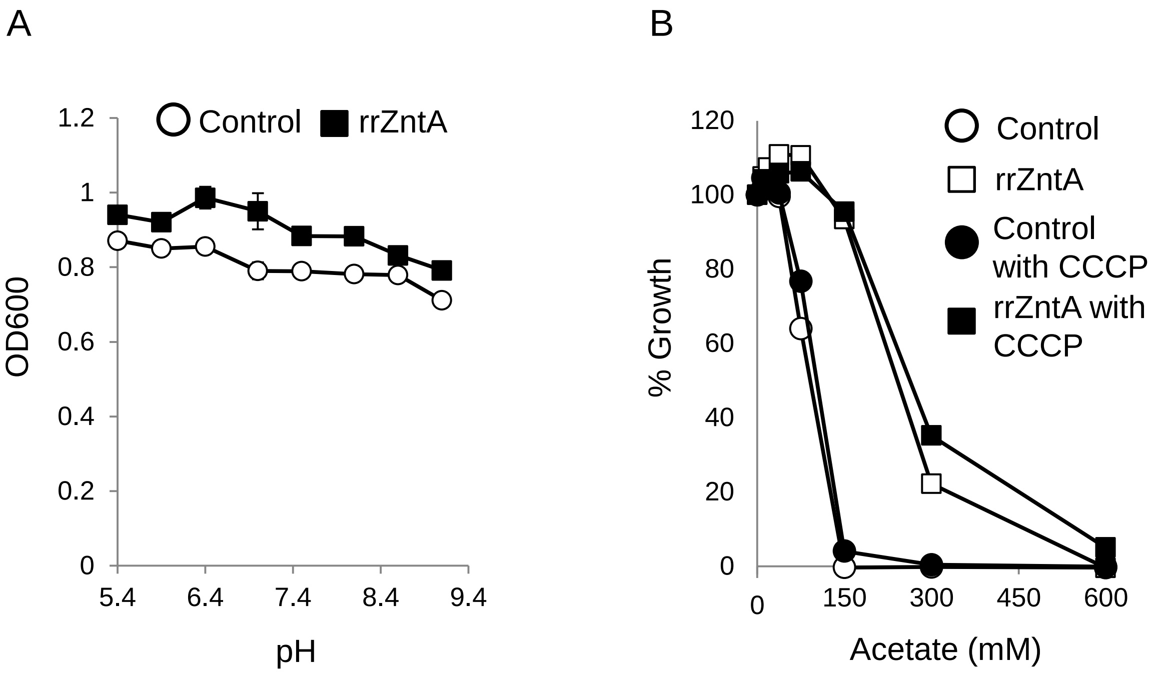

Supplementary Figure 2

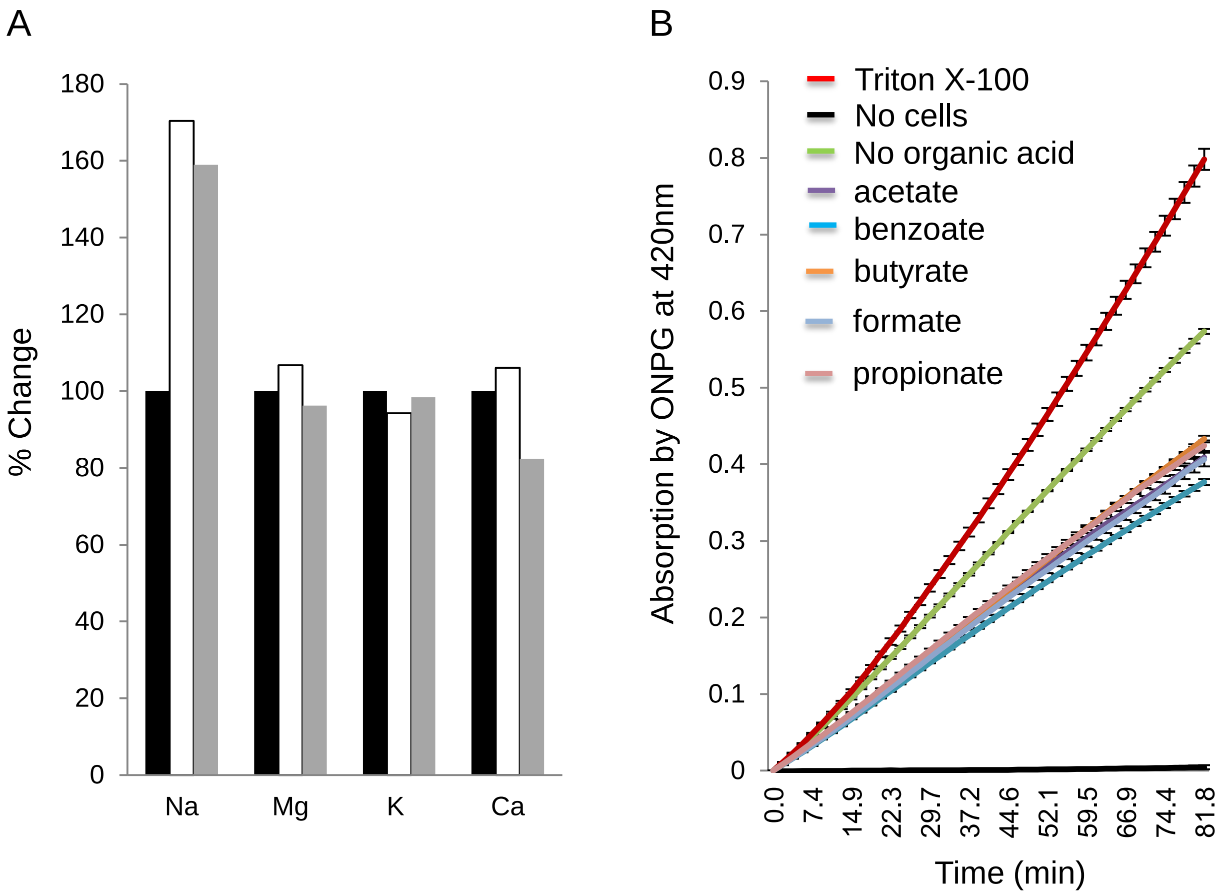

Supplementary Figure 3

A

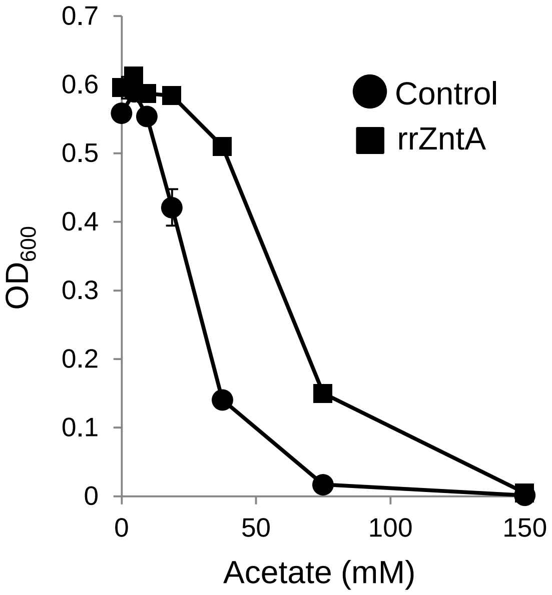

Supplementary Figure 4

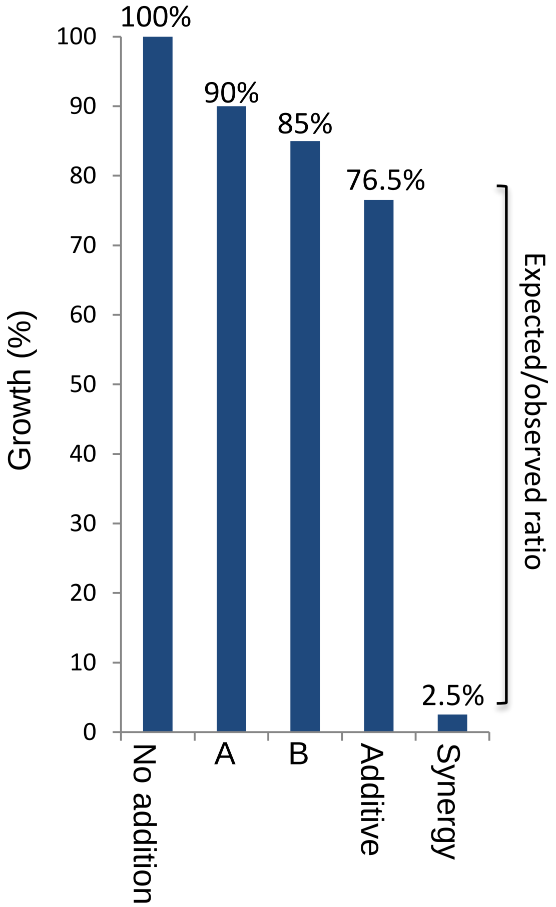

Supplementary Figure 5

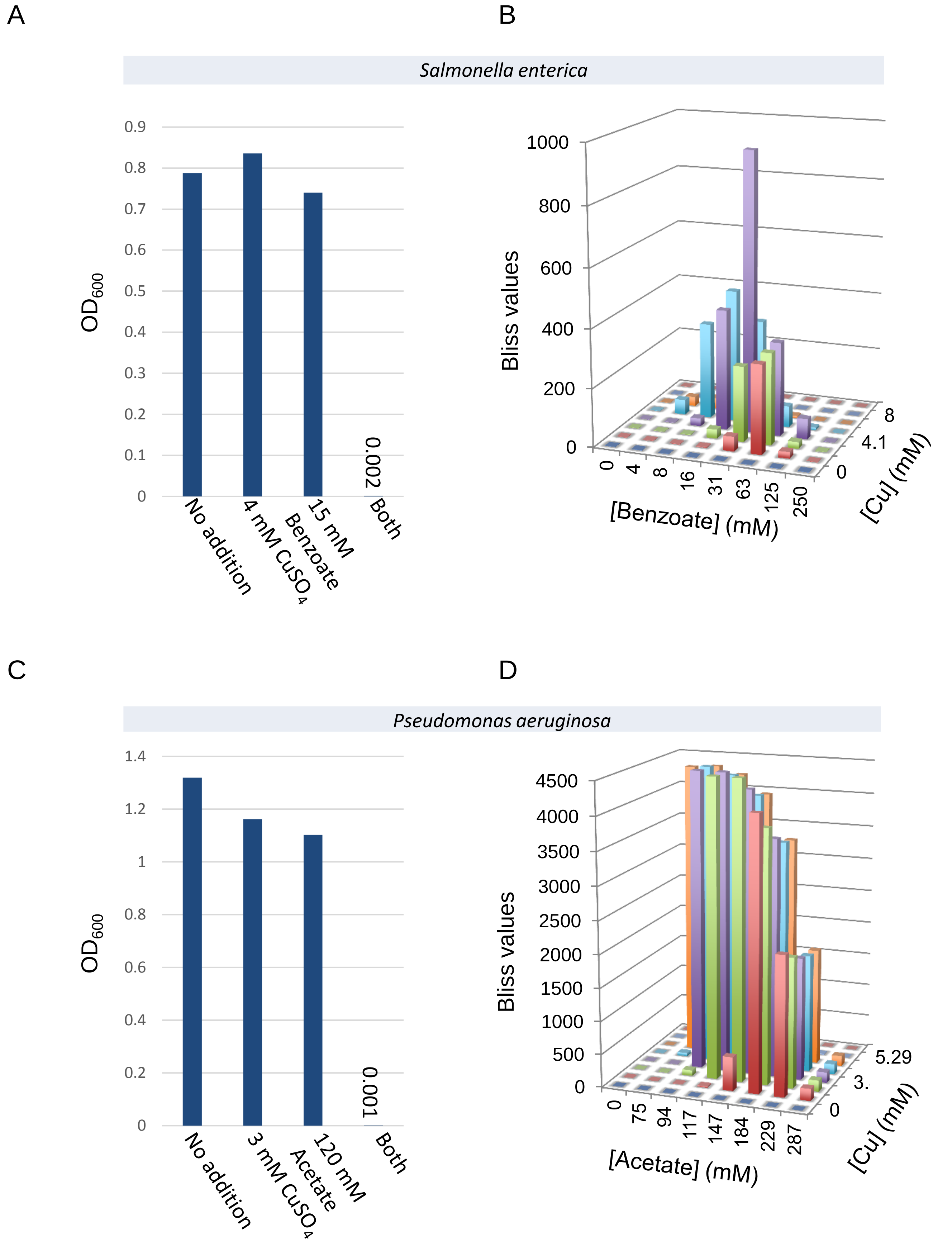

Supplementary Figure 6

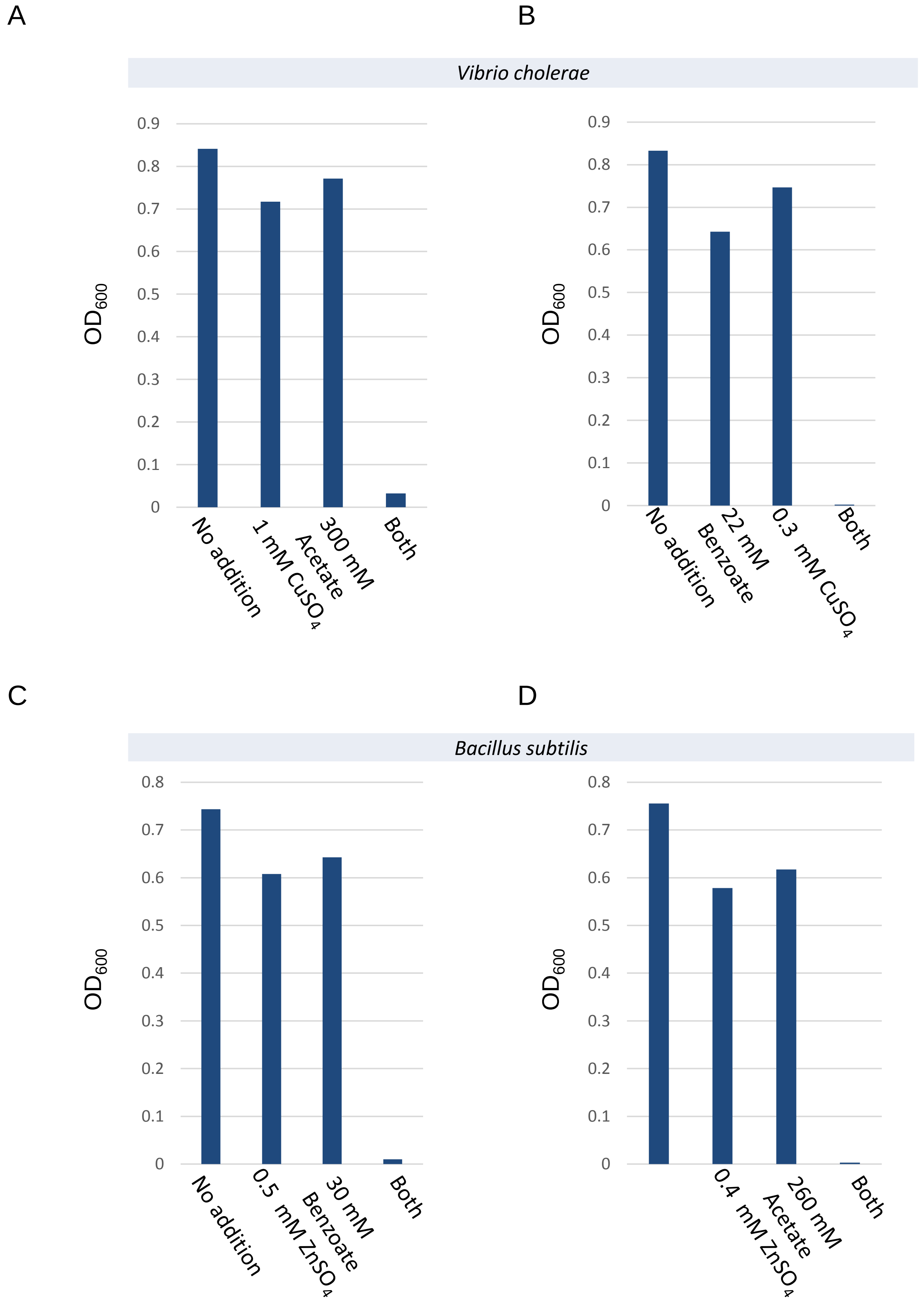

Supplementary Figure 7

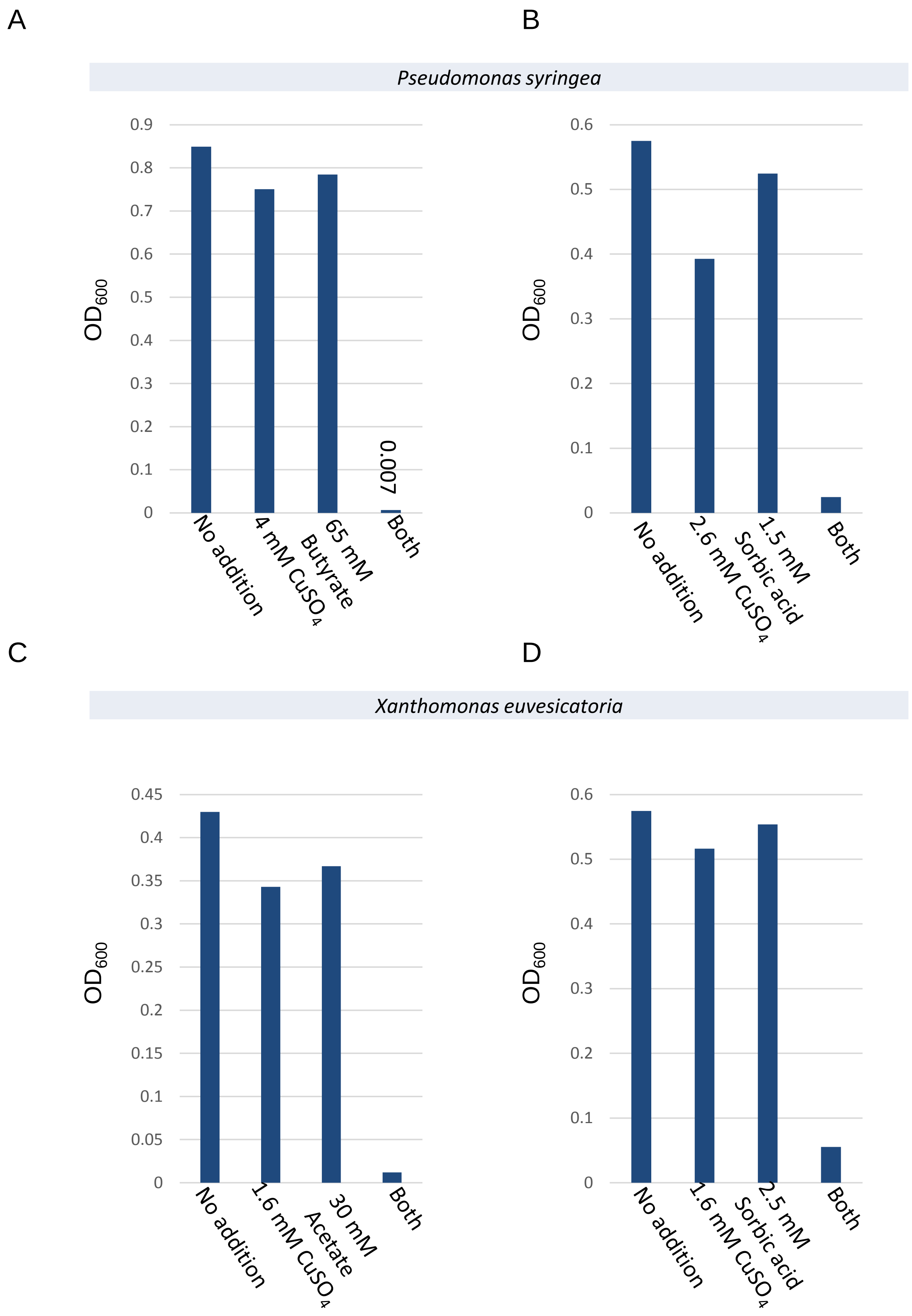

Supplementary Figure 8
